# Giant electron-hole transport asymmetry in ultra-short quantum transistors

**DOI:** 10.1038/ncomms15491

**Published:** 2017-05-31

**Authors:** A. C. McRae, V. Tayari, J. M. Porter, A. R. Champagne

**Affiliations:** 1 Department of Physics, Concordia University, 7141 Sherbrooke Street West, Montréal, Québec, Canada H4B 1R6

## Abstract

Making use of bipolar transport in single-wall carbon nanotube quantum transistors would permit a single device to operate as both a quantum dot and a ballistic conductor or as two quantum dots with different charging energies. Here we report ultra-clean 10 to 100 nm scale suspended nanotube transistors with a large electron-hole transport asymmetry. The devices consist of naked nanotube channels contacted with sections of tube under annealed gold. The annealed gold acts as an *n*-doping top gate, allowing coherent quantum transport, and can create nanometre-sharp barriers. These tunnel barriers define a single quantum dot whose charging energies to add an electron or a hole are vastly different (*e*−*h* charging energy asymmetry). We parameterize the *e*−*h* transport asymmetry by the ratio of the hole and electron charging energies *η*_e−h_. This asymmetry is maximized for short channels and small band gap tubes. In a small band gap device, we demonstrate the fabrication of a dual functionality quantum device acting as a quantum dot for holes and a much longer quantum bus for electrons. In a 14 nm-long channel, *η*_e−h_ reaches up to 2.6 for a device with a band gap of 270 meV. The charging energies in this device exceed 100 meV.

## Abstract (web summary)

By utilizing electron-hole asymmetry in ultra-short single-walled carbon nanotube (SWCNT) transistors, McRae *et al*., develop ‘two-in-one' SWCNT quantum devices that can switch from behaving as quantum-dot transistors for holes to quantum buses for electrons by changing the transistor's gate voltage

An important feature of ultra-clean (intrinsic) single-walled carbon nanotube (SWCNT) channels is that a small gate voltage can tune them from *n*-type (electron-doped) to *p*-type (hole-doped) devices. A large electron-hole (*e*−*h*) transport asymmetry would enable dual functionality quantum SWCNT transistors with drastically different characteristics under electron or hole doping. Unfortunately, the intrinsic transport properties of SWCNTs are mostly *e*−*h* symmetric^1^,^2^ and thus the two types of transport are redundant. Nevertheless, quantum SWCNT devices can be engineered to create an *e*−*h* transport asymmetry. This is achieved for instance in SWCNT quantum dot (QD) transistors whose tunnel barrier heights depend on whether the channel is *n*- or *p*-doped. Such a transport asymmetry has been demonstrated in ultra-clean devices with channel lengths ranging from a few hundreds to 100 nm^3^,^4^. Downsizing ultra-clean *e*−*h* asymmetric SWCNT transistors to 10 nm would create QDs, which can be toggled between two vastly different charging energies. They would be useful to explore fundamental nano-electro-mechanical system (NEMS) physics^5^,^6^,^7^,^8^,^9^ and qubits^2^,^10^,^11^,^12^ at energy scales close the ones in single molecules^13^,^14^ or to create THz bolometers^15^, which are sensitive to two different wavelength ranges (one for each *E*_C_). In addition, a large *e*−*h* transport asymmetry in SWCNT transistors could allow them to act as both active logic elements (QDs) for hole doping and interconnects (quantum bus) for electron doping. This dual functionality is needed to create reconfigurable logic circuits which can be programmed with gate voltages^16^ and would support the development of commercial SWCNT electronics^17^.

Here we demonstrate the capability to engineer a giant *e*−*h* charging energy and conductance asymmetry in ultra-clean (single QD or ballistic) suspended SWCNT channels whose lengths range down to 14 nm. Ultra-clean SWCNTs used to explore many-body physics and quantum bits have previously been limited to channel lengths above 100 nm^3^,^7^,^10^,^11^,^12^,^18^,^19^,^20^. The key feature of our fabrication is to use an annealed-gold film as an electrostatic gate directly deposited on a SWCNT (no dielectric spacer). This *n*-dopes the gold-covered SWCNT sections, which then act as contacts to the naked channel. These gold gates create extremely sharp barriers at the contact-to-channel interfaces (few nanometres wide) and these barriers have different heights whether the channel is *n*- or *p*-doped. We measured transport in five devices under both electron and hole doping of their channels, and at temperatures ranging from 1.3 to 295 K. In a small gap SWCNT device (Device A), *E*_C_'s for electrons and holes differ by orders of magnitude. When this device's channel is *p*-doped, it forms a 102 nm-long low-disorder QD. When the channel is *n*-doped, the device becomes a one-dimensional waveguide where carriers can travel ≈330 nm without losing their phase coherence. In the four devices whose band gaps *E*_g_≳200 meV, the charging energy asymmetry ratio *η*_e−h_ of the charging energies for holes, 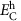, and electrons, 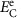 ranges from 1.5 to 2.6. We demonstrate that when we bias appropriately these QDs, a modest gate voltage switches their charge transport from QD to quasi-ballistic. We show that the *e*−*h* charging asymmetry scales inversely with channel length and the SWCNT's band gap. The giant *e*−*h* asymmetry allows us to very significantly increase 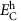, while reducing 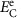, for a given channel length and band gap. The charging energies of our shortest device (Device B) exceed 100 meV, demonstrating the potential for room temperature operation.

## Results

### Ultra-short suspended SWCNT-QDs with ballistic contacts

**Figure 1:**
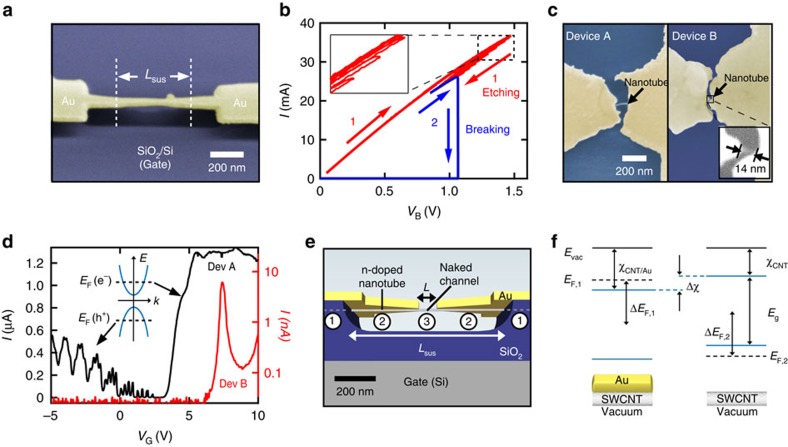
Ultra-short suspended nanotube QD transistors. (**a**) Tilted SEM image showing a suspended gold breakjunction fabricated on top of a SWCNT. The breakjunction is suspended over a length *L*_sus_≈350 nm. The back plane (blue) is used as a global back gate. (**b**) The *I*−*V*_B_ EM data for a gold-on-SWCNT breakjunction at *T*=4 K, showing the process to (1) narrow the junction and (2) create a naked nanotube channel. (**c**) SEM images of Devices A and B after EM. The naked channels are visible and their lengths are *L*_SEM_=111 and 14 nm, respectively. (**d**) *I*−*V*_G_ transistor data from Devices A (black) and B (red), at *T*=1.3 K and *V*_B_=10 mV. We measured a much higher conductance for positive *V*_G_. This indicates that the suspended gold, annealed during the EM, *n*-dopes the underlying tube. (**e**) Geometry of our suspended SWCNT transistors. The labels 1, 2 and 3 refer respectively to the disordered on-substrate nanotube sections, the *n*-doped gold-covered suspended SWCNT sections and the naked SWCNT channel. (**f**) The electronic bands in the gold-covered (left) and naked (right) suspended tube sections. The quantity *E*_g_ is the band gap. The quantities *E*_F,1_, *E*_F,2_, Δ*E*_F,1_ and Δ*E*_F,2_ are the Fermi energies and the Fermi energy shifts from the center of the band gaps in the gold-covered and naked nanotube, respectively. *χ*_CNT/Au_ and *χ*_CNT_ are the electron affinities in the gold-covered and naked sections, whereas Δ*χ* is the electron affinity difference between the two.

In Table 1, we list for the five studied devices, the following key parameters: band gap *E*_g_ as extracted via QD transport, channel length *L*_SEM_ measured via scanning electron microscope (SEM) imaging, channel length *L*_G_ extracted from QD transport data and ratio *η*_e−h_ of the charging energies for holes, 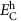, and electrons, 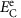. Figure 1 summarizes the fabrication and contact geometry of our suspended SWCNT devices^5^,^21^. We first fabricated suspended gold-on-SWCNT breakjunctions (see Methods). Figure 1a shows a breakjunction and *L*_sus_ indicates the length over which the gold is suspended. The substrate (blue) is used as a global back-gate electrode. The final fabrication step is to create a nanometre-long naked SWCNT channel in the centre of the gold breakjunction. To do so, we used a previously reported feedback-controlled electromigration (EM) procedure^5^,^22^ summarized in Fig. 1b (see Methods), where a bias voltage, *V*_B_, is applied between the source and drain gold electrodes. This EM process exposes a short naked SWCNT channel. Figure 1c shows top-view SEM images of Devices A and B whose channel lengths are 111±5 and 14±3 nm, respectively. Figure 1d shows the current-gate voltage, *I*−*V*_G_, characteristics at *T*=1.3 K for Devices A (black) and B (red). The EM not only uncovers short naked SWCNT channels but also anneals them such that the QDs in the channels are nearly undoped^5^ with their charge neutrality point close to *V*_*G*_=0. Close inspection of the gold surface (Supplementary Fig. 1) reveals that the suspended portion of the gold film changes texture after the EM. The inset of Fig. 1d shows the Fermi level position in the channel under electron or hole doping. The current (conductance) is much higher when the back gate *n*-dopes rather than *p*-dopes the channel. This is consistent with the suspended gold film acting as a top gate which n-dopes the SWCNT sections it covers, and that these nanotube sections act as contacts to the channel (see Fig. 1e). We emphasize that we use pure gold films, without any adhesion layer, to create clean SWCNT-Au interfaces. Gold's workfunction is very close to the threshold, ≈5.4 eV, where physisorbed metal films switch from *n*-doping to *p*-doping graphene^23^ and SWCNTs^24^,^25^. A small change in gold's workfunction due to the removing of adsorbed oxygen on the gold during the EM can tune the gold films from *p*- to *n*-doping^24^,^26^. We observed this effect in Device B (Supplementary Fig. 2). We note that the fabrication process of our ultra-clean SWCNT devices is compatible with the optical measurement of the SWCNT chirality^27^,^28^ before the gold deposition. Such ultra-clean SWCNT transistors with a known tube chirality could lead to breakthroughs in SWCNT physics^2^.

The electronic interactions between an ultra-clean SWCNT and gold are weak^29^, and the density of states in the SWCNT is unaffected by the gold film. This is because SWCNT electronic wavefunctions have large wavevectors and the conservation of momentum suppresses the tunnelling matrix elements between these high-momentum states and gold states^30^. It follows that the injection length for an electron to tunnel between gold and the SWCNT is very long (μm)^29^,^31^. Electrostatic disorder leads to stronger SWCNT–gold interactions and shorter injection lengths^29^,^30^. This knowledge is necessary to describe the electron transport in the gold covered sections of our SWCNTs and the device design shown in Fig. 1e. The gold film covering the SWCNT sections is thermally anchored to the substrate and not annealed during the EM process. This leaves the sections acting as diffusive (disordered) contacts where the gold-SWCNT coupling is stronger^29^ and where most of the electrons are injected from gold into the SWCNT^19^,^32^,^33^,^34^. The suspended gold film covering the tube sections labelled is annealed as evidenced from both transport data (Fig. 1d) and the film texture (Supplementary Fig. 1). Finally, the naked SWCNT channel labelled is thoroughly annealed by the EM process^5^,^21^ and can be doped with either holes or electrons via *V*_G_. The data presented below will demonstrate that the charge transport is ballistic in both sections and of the SWCNTs, and that *e*−*h* asymmetric tunnel barriers form where the two sections connect.

The left hand side of Fig. 1f shows an example of the band diagram for the SWCNT contacts (sections ) and the right hand side shows the bands in the naked channel (section ). The dashed lines indicate the positions of the Fermi energies *E*_F,1_ and *E*_F,2_ in each section and their shifts Δ*E*_F,1_ and Δ*E*_F,2_ away from the centre of the band gap. The band gap has the same value in both SWCNT sections, as they belong to the same tube. The nanotube's electron affinity, *χ*_CNT_, is defined as the energy between the bottom of the conduction band and the vacuum energy, *E*_vac_. On the left side of Fig. 1f, gold transfers charges to the SWCNT and moves *E*_*F*,1_ up from the centre of the gap. The exact amount of doping varies depending on the crystalline orientation of the gold, as well as the quality of annealing (oxygen content). The relevant values of Δ*E*_F,1_ for our devices are reported to range from 0.05 to 0.2 eV^24^,^25^,^31^,^35^,^36^ and we use a median value of 0.12 eV to draw our band diagrams. The gold film can also modify the nanotube's electron affinity by Δ*χ*. This shift is expected to be 0.03–0.05 eV^25^,^35^ and we use 0.05 eV to draw the bands in our devices. These parameters correctly predict several features of the data below, such as the presence or absence of a barrier, the sign of the *e*−*h* charging energy asymmetry and correlate with the magnitude of the asymmetry. Moreover, the discussion and conclusions below remain valid over the range of reported Δ*E*_*F*,1_ and Δ*χ* values. The Fermi energy of the naked SWCNT channel, *E*_F,2_, can be tuned using *V*_G_. The gating efficiency of the back gate (dielectric of 130 nm vacuum plus 170 nm SiO_2_) is much weaker than the gating efficiency of the gold film (top gate), which is only 3 Å away from the SWCNT^25^. This means that the back gate does not significantly affect *E*_F,1_, but only effectively tunes *E*_F,2_. This is confirmed by the transport data below showing that the lengths (confinement) of the SWCNT-QDs are independent of *V*_G_. At the junctions between the tube sections and , the band diagrams in Fig. 1f are brought into contact and equilibrate to form homojunctions.

**Figure 2:**
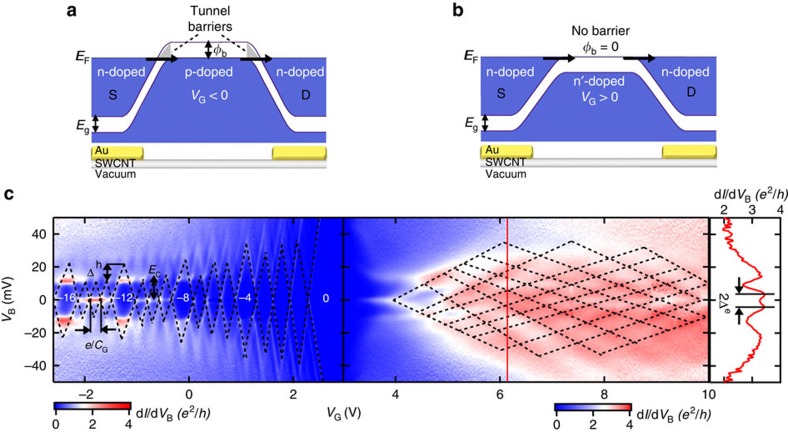
The *e*−*h* transport asymmetry and ballistic contacts. (**a**,**b**) Schematics of the bands at the junctions between the *n*-doped (gold-covered) nanotube contacts and naked channel in Device A (*E*_g_=28 meV) when the channel is (**a**) *p*-doped and (**b**) *n*-doped. Band-to-band tunnel barriers (shaded triangles) of height *φ*_B_=*E*_g_ form between the nanotube contacts and the *p*-doped channel. On the other hand, when the naked channel is *n*-doped *φ*_B_=0. (**c**) Charge transport data (*dI*/*dV*_B_−*V*_B_−*V*_G_) for Device A at *T*=1.3 K. The charge neutrality point of the channel is clearly visible around *V*_G_=3 V. A striking asymmetry is visible in the transport data between hole doping (*V*_G_<3 V) and electron doping (*V*_G_>3 V) of the channel. For hole doping, a fourfold Coulomb diamond structure indicates a single QD. The gate-to-QD capacitance *C*_G_ corresponds to a channel length of 102±5 nm, closely matching *L*_SEM_ in Fig. 1c. Under electron doping, the transport data show clear Fabry–Pérot quantum interferences (see vertical outset). The spacing between conductance maxima in *V*_B_ is 5±1 mV, giving *L*_FP_=330±70 nm. This matches the suspension length of the gold film *L*_sus_=350±70 nm (Supplementary Fig. 1). It confirms that the transport in the suspended gold-covered nanotube sections is ballistic and preserves the quantum phase of the electrons travelling through the channel.

Figure 2a,b show schematics of the homojunctions (Supplementary Note 4) in Device A, *E*_g_=28 meV. Figure 2a shows the band alignment when the channel is hole doped, whereas Fig. 2b shows the electron doping configuration. Tunnel barriers form at the contacts when the channel is *p*-doped. The transport mechanism across the barriers in Fig. 2a is band-to-band tunnelling and has been previously described for SWCNT transistors^3^,^37^. Approximating the shape of the bands at the homojunctions using the Wentzel-Kramers Brillouin (WKB) model^38^ (shaded triangular regions), the tunnel barrier height is *φ*_B_=*E*_g_=28 meV. When the channel is electron doped (Fig. 2b), the charge transport only involves the conduction band and there is no tunnel barrier. Low-temperature (*T*=1.3 K) transport data from Device A are shown in Fig. 2c, where the colour scales show the differential conductance *dI*/*dV*_B_ as a function of *V*_G_ and bias voltage *V*_B_. The negative (positive) integer labels *N* indicate the number of holes (electrons) in the SWCNT channel. Under hole-doping of the channel, clear Coulomb blockade diamonds indicate QD transport. When the channel is electron doped, transport data along the *V*_G_ and *V*_B_ directions show strong Fabry-Pérot interferences characteristic of ballistic transport^32^,^34^. The dramatic asymmetry in quantum transport between holes (QD) and electrons (ballistic channel) is a consequence of the different contact barriers for the two types of doping. A closer look at the data in Fig. 2c will confirm the device geometry shown in Fig. 1e.

On the left hand side of Fig. 2c, we observe four-fold quantum degeneracy^2^ of the hole-doped QD energy levels. We extract an energy level spacing for holes of Δ^*h*^≈12±1 meV (height difference between neighbouring tall and short diamonds) and a charging energy 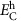
=11±1 meV (height of short diamonds). The width of the Coulomb diamonds for holes is 0.21±0.01 V=*e*/*C*_G_, where *C*_G_ is the capacitance between the QD and back gate. We obtain a QD length, *L*_G_=102±5 nm, from the measured *C*_G_ using a wire over a plane capacitor model (Supplementary Note 3). This length closely matches the length of the channel as measured by SEM, *L*_SEM_=111±5 nm. We note that on average in our samples *L*_G_<*L*_SEM_ is expected due to the two finite width *p*–*n* or *n*–*n*' junctions at each end of the QD. Comparing the measurements of *L*_G_ and *L*_SEM_ in all devices (Table 1), we find an average *p*–*n* junction length of *L*_pn_=3±1 nm. An independent estimate of *L*_pn_ is obtained from the SWCNT screening length^29^, 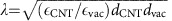, where 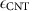
≈10 is the SWCNT permittivity^39^, *d*_CNT_∼1.3 nm is the tube diameter, 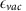
=1 is the permittivity of vacuum and *d*_vac_≈0.3 nm^25^,^40^ is the thickness of the vacuum dielectric between the tube and gold. This gives *λ*≈2 nm, matching the *L*_pn_ extracted above. It is remarkable that we can think of the suspended (annealed) gold film as a gate electrode for the SWCNT, with a dielectric spacer of 3 Å, offering the possibility to create extremely sharp *p*–*n* junctions. This capability will find applications in testing the ultimate downscaling of SWCNT transistors^21^,^41^ and creating nanometre-sized phase coherent electronic devices^2^,^12^,^42^,^43^ and NEMS^5^,^6^,^7^,^8^,^9^.

Although the suspended gold gates in Device A confine holes to a QD of length *L*_G_=102 nm, they do not confine electrons inside the naked channel. Using the transport data in the vertical line cut of Fig. 2c, we measure an energy level spacing Δ^e^=*e*Δ*V*_B_=5±1 meV between the interference maxima. We extract the length of the electron cavity as *L*_FP_=*hv*_F_/(2Δ^e^)=330±70 nm, where *v*_F_=8 × 10^5^ m s^−1^ is the Fermi velocity. This cavity length matches the suspension length of the gold gates, *L*_sus_≈350±70 nm (Fig. 1e and Supplementary Fig. 1). The coherent Fabry-Pérot (FP) interferences and cavity length confirm that the majority of the current in the contact sections flows through the SWCNT and not the gold film. This is expected due to the long charge injection length in ultra-clean SWCNTS^29^,^31^,^40^. The FP interferences also imply that the annealed gold covering sections does not prevent ballistic transport in the underlying SWCNT^44^ and we do not detect any backscattering at the interfaces between sections and . Thus, the naked channel is very nearly ballistic and we extract a very conservative upper bound for its charging energy from the longer FP cavity. Using an open QD model^34^ for the cavity formed by sections 2 and 3 together, we extract an approximate electron charging energy of 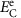
∼0.1 meV, giving a charging energy asymmetry ratio 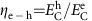
≳100. We note that previously reported SWCNT devices, which showed QD to FP *e*−*h* asymmetry^19^,^33^,^45^ were not fabricated with suspended and annealed gold films, and thus had equal channel lengths for holes and electrons. The important new information in Fig. 2c are that the annealed gold film acts as a local top gate creating ballistic SWCNT contacts and a gate programmable QD-to-ballistic transistor with vastly different channel lengths under electron and hole doping. Specifically, applying a small *V*_G_ to Device A toggles between a 102 nm QD and a 330 nm ballistic wire. In the following section, we demonstrate in SWCNT-QD transistors, whose *L*_G_ ranges down to 7 nm and *E*_g_ up to 270 meV, that a large *e*−*h* transport asymmetry creates QDs with two *E*_C_'s.

### Giant *e*−*h* transport asymmetry in 200 meV band gap SWCNT-QDs

For room-temperature applications and to explore many-body QD physics, we would like to extend the ability to create large *e*−*h* transport asymmetry to SWCNT transistors whose *E*_g_'s are larger than in Device A. We studied four larger *E*_g_ devices whose naked channels host single QDs whether they are doped with holes or electrons. The heights of the QD contact barriers for the two doping configurations are vastly different and lead to two different *E*_C_'s.

**Figure 3:**
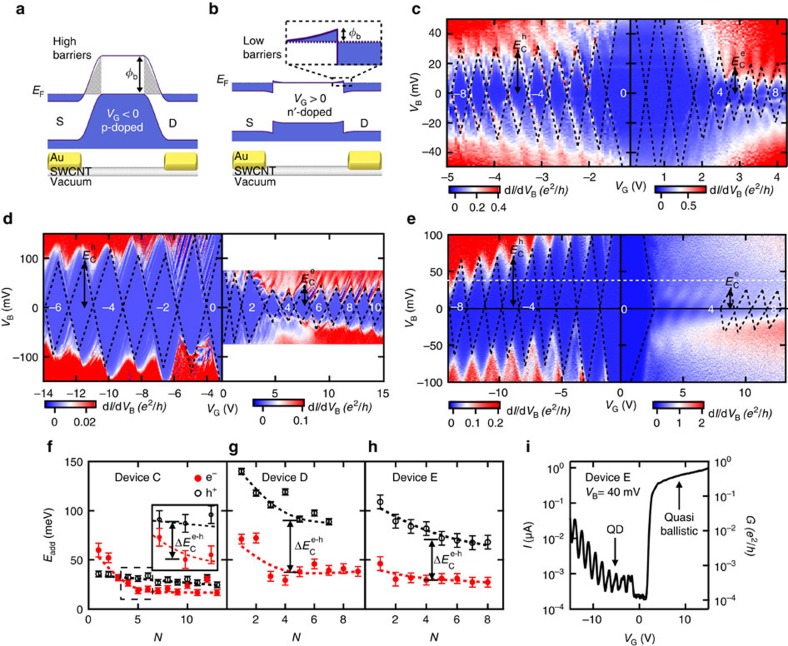
Giant *e*−*h* charging energy asymmetry in 200 meV band gap nanotube QDs. (**a**,**b**) Schematics of the junctions between the *n*-doped nanotube contacts and naked tube channel in Device C, *E*_g_=190 meV. (**a**) When the channel is *p*-doped, the band-to-band tunnel barriers (shaded triangles) have a height *φ*_B_=190 meV. (**b**) When the channel is *n*-doped, the tunnel barriers are much smaller, *φ*_B_∼12 meV (Supplementary Note 4). (**c**–**e**) *dI*/*dV*_B_−*V*_B_−*V*_G_ transport data for Devices C, D and E, respectively (*T*=4.0, 1.3 and 50 K). In all devices, clear Coulomb diamonds are visible for both hole and electron channel dopings and show the formation of a single QD in the channel. The charging energies, that is, heights of the odd-labelled diamonds, are much larger for holes than for electrons due to the tunnel barrier asymmetry. (**f**–**h**) Extracted addition energy, *E*_add_=height of diamonds, versus charge number, *N*, for both holes (open black data) and electrons (filled red data) in Devices C, D and E. The data for odd *N*, for which *E*_add_=*E*_C_ are interpolated with dashed lines. The charging energies decrease with increasing *N* and a clear offset, Δ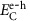 is visible between hole and electron data. The value of Δ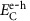 becomes roughly constant at large *N*. The errors bars represent the uncertainty in the extracted *E*_add_ and stem from the limited resolution of the Coulomb diamond heights in **c**–**e**. (**i**) *I*−*V*_G_ data for Device E at *V*_*B*_=40 meV (along white dashed line in **e**).

Figure 3a,b show the homojunctions forming at the interfaces between the contacts and channel in Device C. When the channel is hole doped (Fig. 3a), tall tunnel barriers of *φ*_B_≈*E*_g_=190 meV form where the contacts (sections ) meet the SWCNT channel (sections ). Figure 3b shows the configuration when the channel is electron doped. The transport across the *n*–*n*' junctions only involves the conduction band, and the barriers are much smaller, *φ*_B_≈12 meV. This *e*−*h* barrier height asymmetry creates an *e*−*h* asymmetry of the source-QD and drain-QD capacitances, *C*_S_ and *C*_D_, respectively. It follows that the QD charging energy *E*_C_=*e*/(*C*_S_+*C*_D_+*C*_G_)=*e*/*C*_Σ_, depends on whether the QD is populated with holes or electrons. This is visible in Fig. 3c–e where *dI*/*dV*_B_−*V*_B_−*V*_G_ data for Devices C, D and E are shown. The Coulomb diamond heights in Fig. 3c–e, that is, the addition energies *E*_add_=*E*_C_+Δ, are much larger for holes (left) than for electrons (right).

We use the data in Fig. 3 to quantify both the *e*−*h* charging energy asymmetry Δ
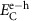
=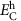
−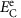 and the asymmetry ratio *η*_e−h_=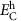/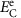. We extract from Fig. 3c–e the heights of the diamonds as a function of the charge occupation number *N* as shown in Fig. 3f–h. The dashed lines are interpolations of the odd-*N* data points, for which *E*_add_=*E*_C_, as Devices C, D and E show a twofold degenerate energy spectrum. We note that Δ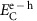 converges to a roughly constant value at large *N*. To compare the asymmetry between devices, we extract *η*_e−h_ at *N*=5. The *η*_e−h_ in Devices C, D and E are 1.5, 2.1 and 2.5, respectively (Supplementary Note 5). Although the *E*_g_ for all three of these devices are comparable, the channels in Devices D and E are three times shorter than in Device C. Both Devices D and E have significantly larger *η*_e−h_ than Device C and this correlation is also confirmed by Device B (Supplementary Note 2). We thus conclude that *η*_e−h_ scales inversely with length. This explains why we can observe large *η*_e−h_ in ultra-short QDs whose band gaps range up to 270 meV, while previous experiments^1^ on much longer QD devices (few 100 s of nm) showed *η*_e−h_≈1 in devices with similar band gaps. Figure 3i shows *I*−*V*_G_ data for Device E at *V*_*B*_=40 meV. We observe that similarly to Device A (Fig. 2c), it is possible to toggle this large band gap, *E*_g_=170 meV, and very short, *L*_G_=15 nm, device from behaving as a QD to a nearly ballistic bus by applying modest *V*_G_. To further understand the length dependence of *η*_e−h_, it is useful to analyse the capacitances of the SWCNT-QDs versus *L*_G_.

## Discussion

**Figure 4:**
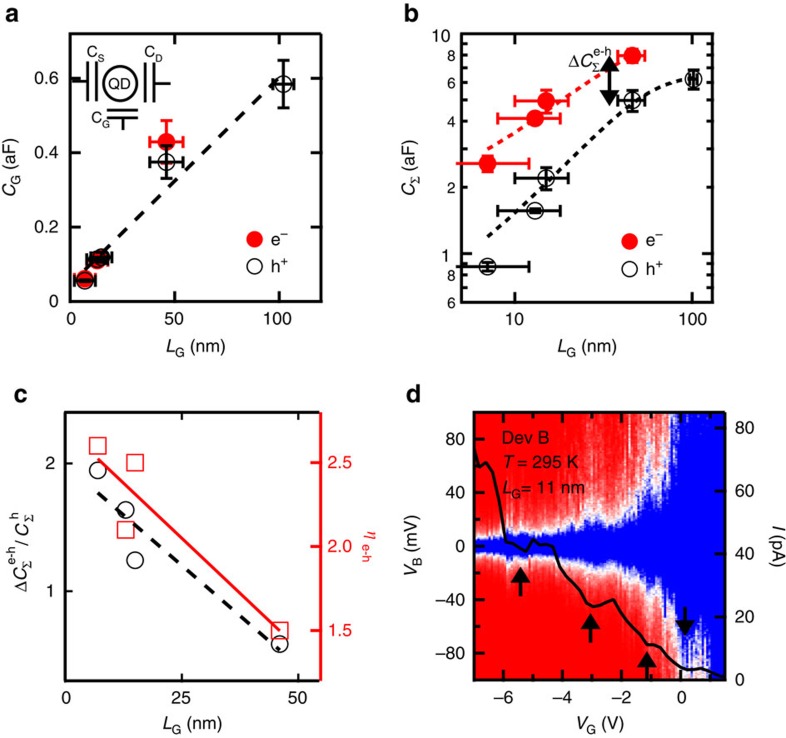
Origin of the *e*−*h* charging energy asymmetry. (**a**) The gate-to-dot capacitance, *C*_G_, in our devices is *e*−*h* symmetric and scales linearly with *L*_G_. The inset cartoon of the QD devices shows the lumped element interpretation of *C*_G_, *C*_S_ and *C*_D_. The error bars represent the uncertainty in the widths of the Coulomb diamonds in the transport data of Fig. 3 and Supplementary Fig. 3. (**b**) The total QD capacitance *C*_Σ_ increases approximately linearly as a function of *L*_G_ in our devices. A clear capacitance offset 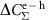 is visible between the hole and electron data due to the *e*-*h* tunnel barrier asymmetry. This offset is at the origin of the *e*−*h* charging energy asymmetry. The error bars represent the uncertainty in the total QD capacitance stemming from the uncertainties in the slopes of the Coulomb diamonds in the transport data of Fig. 3 and Supplementary Fig. 3. (**c**) The relative *e*-*h* capacitance asymmetry 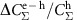 decreases with increasing *L*_G_ and explains why *η*_e-h_=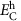/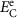 also decreases with increasing *L*_G_. The dashed and solid lines are linear fits of the data. (**d**) *I*−*V*_B_−*V*_G_ data in Device B at 295 K with a superimposed *I*−*V*_G_ data cut, *V*_B_=10 mV. The spacing of the conductance oscillation (black arrows) gives *L*_G_≈11 nm.

In Fig. 4a,b, the data in black (red) are for hole (electron)-doped channels. Figure 4a shows *C*_G_ extracted from the widths of the *N*=5 Coulomb diamonds for all devices versus *L*_G_ (except for Device B, where only *N*=1 is available). *C*_G_ is the same for hole or electron occupations of the QD and ranges from ≈0.05 to 0.6 aF. As expected, *C*_G_ scales linearly with *L*_G_. Figure 4b shows the total QD capacitance *C*_Σ_=*C*_S_+*C*_D_+*C*_G_ extracted from the slopes of the diamonds in Figs 2 and 3 as a function of *L*_G_ (Supplementary Note 2). There is a marked *e*−*h* asymmetry between the total capacitances for holes and electrons, which we label 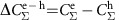. Figure 4c shows that both the relative capacitance asymmetry 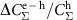 (left axis) and the resulting *η*_e−h_ (right axis) decrease rapidly with *L*_G_ for Devices B, C, D and E, which all have similar band gaps.

We can also isolate the qualitative effect of *E*_g_ on *η*_e−h_. A length change of a factor of three between Devices C, D and E (similar *E*_g_'s) changes *η*_e−h_ by less than a factor of 2. An even smaller length difference between Devices A (Fig. 2c) and C (Fig. 3c), but coupled with an order of magnitude change in *E*_g_, leads to a difference of two orders of magnitude in *η*_e−h_. The change in *η*_e−h_ between Devices A and C can thus be predominantly ascribed to their different *E*_g_'s. This inverse dependence of *η*_e−h_ on *E*_g_ can be understood by inspecting Fig. 2a,b. The relative *e*−*h* barrier height asymmetry decreases rapidly as *E*_g_ increases and becomes larger than the doping Δ*E*_F,1_ induced by the gold gates.

As the values of *E*_C_ and *φ*_*B*_ in our SWCNT-QD transistors are large compared with *k*_B_*T*_room_, we can envision making use of the *e*−*h* transport asymmetry in room temperature QD devices. Figure 4d shows *I*−*V*_B_−*V*_G_ data for Device B at 295 K, with a superimposed *I*−*V*_G_ trace taken at *V*_B_=10 mV. The SEM image of Device B in Fig. 1c shows a channel length of only 14 nm and transport data a bandgap of 270 meV (Supplementary Note 2). During the warm up from 1.3 K to room temperature, Device B was exposed to oxygen (Supplementary Note 1) leading to *p*-doped SWCNT contact sections (Supplementary Fig. 2). In Fig. 4d, remnants of conductance oscillations are visible when the channel is *p*-doped. The spacing of these oscillations matches the *L*_G_ measured at low temperature and suggests the formation of a QD with an 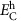
∼25 meV. The data in Supplementary Fig. 2 are consistent with 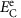
>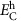 at 295 K and we expect the *e*−*h* asymmetry to survive at room temperature.

In summary, we used suspended annealed gold films as local gates to create *e*−*h* asymmetric suspended SWCNT-QD transistors (Fig. 1e). Using transport measurements, we showed that the gold gates permit ballistic (coherent) charge transport in the underlying SWCNT sections and create nanometre-sharp tunnel barriers at the edge of the SWCNT channels. These barriers were of vastly different heights whether a channel was hole or electron doped. This produced a giant *e*−*h* transport asymmetry in the five SWCNT transistors studied. We used this asymmetry to create two types of SWCNT quantum devices with dual functionality. We first showed a low-*E*_g_ SWCNT device where a small gate voltage could switch the device from being a 330 nm-long quantum bus, under electron doping, to a 102 nm-long QD, for hole doping. Second, we reported four devices with 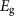
≳200 meV where a small *V*_G_ could switch the QD between two charging energies whose values differed by a factor up to 2.6, or switch the transport from QD to nearly ballistic. The size of the *e*−*h* transport asymmetry in these devices scaled inversely with the length of the channel and the band gap of the tube. In a 14 nm-long channel device, we measured low-temperature charging energies for holes and electrons exceeding 100 and 50 meV, respectively, which suggests that the *e*–*h* asymmetry could survive in room temperature devices. Nanotube transistors with a giant *e*−*h* transport asymmetry could find applications in exploring the physics of near molecular size SWCNT-NEMS^46^, to shrink down SWCNT qubits^12^,^47^ and to create SWCNT THz detectors^15^ or gate programmable transistors^16^.

## Methods

### Lithography

We use *e*-beam lithography to define bow-tie-shaped breakjunctions in a poly(methyl methacrylate) bilayer on SWCNTs deposited on a SiO_2_/Si substrate. We then evaporate a 40 nm-thick gold film (no adhesion layer) to define the gold junctions. We use a wet buffered oxide etch to freely suspend the central portion of the gold breakjunctions as shown in Fig. 1a.

### Electromigration

The red data in Fig. 1b show the first stage of EM where we narrow down the central bowtie-shaped gold junctions. To do so, we ramp up a bias voltage, *V*_B_, across the device and monitor the current, *I*. The bias is applied to the source electrode and the drain is grounded. As the resistance, *R*=*V*_*B*_/*I*, increases, a feedback circuit rapidly ramps down *V*_B_ to avoid an avalanche breaking of the junction. We repeat the feedback controlled-EM process iteratively to gradually narrow down the junction (increase *R*) and these successive voltage ramps are visible in the inset of Fig. 1b. We learned previously that using a continuous *V*_B_ ramp to break a gold junction gives a central junction gap which is proportional to the initial resistance value of the junction^5^,^21^,^22^. Thus, we stop the narrowing of the gold junction at a desired initial resistance to achieve a target channel length. We then proceed to step two of the EM (blue data), where the junction is broken with a single continuous voltage ramp.

### Band gap measurement

To determine the band gaps of the tubes in our devices, we use the data at the charge neutrality point *N*=0, corresponding to the largest blockade diamond in Figs 2c and 3c–e, and Supplementary Fig. 3. The band gap is given by 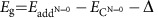 where 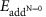 is the height of the *N*=0 diamond, *E*_C_ is the charging energy and Δ is the single-particle energy spacing. See Supplementary Note 2 for details.

### Data availability

The data that support the findings of this study are available from the corresponding author upon request.

## Additional information

**How to cite this article:** McRae, A. C. *et al*. Giant electron-hole transport asymmetry in ultra-short quantum transistors. *Nat. Commun.* **8,** 15491 doi: 10.1038/ncomms15491 (2017).

**Publisher's note**: Springer Nature remains neutral with regard to jurisdictional claims in published maps and institutional affiliations.

## Supplementary Material

Supplementary Information Supplementary Figures, Supplementary Tables, Supplementary Notes and Supplementary References

## Figures and Tables

**Table 1:** Key parameters for the five SWCNT-QD transistors reported.

**Device**	***E***_**g**_ **(meV)**	***L***_**SEM**_ **(nm)**	***L***_**G**_ **(nm)**	***η***_**e−h**_
A	28±5	111±5	102±5	Above 100
B	270±50	14±3	7±5	2.6
C	190±20	42±7	46±8	1.5
D	250±20	16±4	13±5	2.1
E	170±50	24±8	15±5	2.5

*E*_g_ is the band gap extracted from transport data, *L*_SEM_ the channel length measured via SEM, *L*_G_ the channel length from transport data and *η*_e−h_=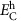/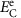 is the measured charging energy asymmetry between electron and hole doping of the quantum dots.
