# Supplementary material for: Giant electron-hole transport asymmetry in ultra-short quantum transistors
Source: Nat Commun. 2017 May 31;8:15491. doi: 10.1038/ncomms15491 (PMC5460015; doi:10.1038/ncomms15491)
Supplement: Supplementary Information — Supplementary Figures, Supplementary Tables, Supplementary Notes and Supplementary References [file ncomms15491-s1.pdf]

Supplementary Note 1:

Suspended gold gates: length, annealing and charge transfer to SWCNTs

1.1 Length of the gold gates (suspension length of breakjunctions)

The gold breakjunctions, as shown in Fig. 1a, are suspended using a buffered oxide etch (BOE) which removes the SiO_2 underneath the central portion of the bowtie-shaped junctions. We determine the lengths of these suspended gold breakjunctions (acting as gold gates) via tilted SEM imaging, as shown in the inset of Supplementary Fig. 1a. We measured the suspension length, L_{sus}, of 19 samples prepared with the same BOE etching recipe as for our reported devices. The results are shown in Figure S1a. The mean suspension length is L_{sus}= 350 ± 70 nm.

We corroborate the suspension lengths of our devices by investigating the texture of the Au film on top of the SWCNT as shown in Supplementary Fig. 1b. During the gold electromigration (EM), the portion of the gold film which is substrate-supported is heat-sunk and not annealed. This substrate-supported gold film shows a standard polycrystalline texture, as shown in inset 1 of Supplementary Fig. 1b. During the EM, Joule heating raises the temperature of the suspended gold to a few hundred degrees Celsius [S1] and anneals the gold into a more uniformly textured film as shown in inset 2 of Supplementary Fig. 1b. The boundaries of this gold texture change are shown in the main panel of Supplementary Fig. 1b (Device A) with dashed red lines. Using these boundaries to measure the suspension length, we find a good agreement with the length from tilted SEM.

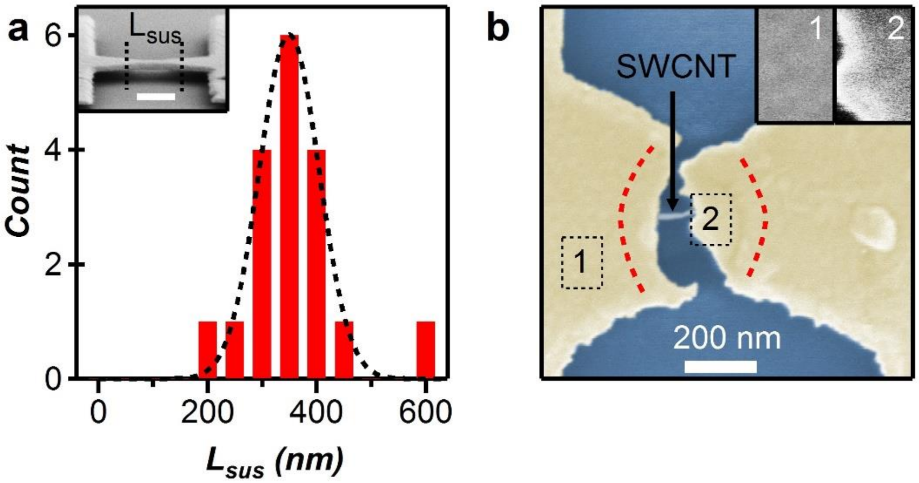

Supplementary Figure 1. Length of the gold gates (suspension length of breakjunctions). **a.** Histogram of 19 suspended devices with BOE etching depths of 130 to 143 nm (closely matching the parameters for our reported devices). The mean suspension length is L_{sus} = 350 ± 70 nm. Inset: 75º tilted SEM image of a device, scale bar is 300 nm. **b.** False colored top-view SEM image of Device A, showing not annealed

(1) and annealed (2) sections of the film corresponding to the on-substrate and suspended sections of the breakjunction. Insets 1 and 2 correspond to the boxes in the main panel and show the film texture after the EM, for the on-substrate and suspended gold, respectively.

1.2 Charge transfer from gold to SWCNTs: n-type to p-type

The suspended and annealed gold film described in section 1.1 plays an important role in the transport behaviour of our devices. This film acts as a gate which dopes the SWCNT tube sections it covers. These doped SWCNT sections act as contacts to the naked SWCNT channels (see Fig. 1e). While gold normally *p*-dopes SWCNTs, it is well documented that annealing gold removes oxygen from the film and changes its work function [S2] such that it becomes *n*-doping. Supplementary Figure 2a shows transport data from Device B after annealing (suspended gold is *n*-doping), and Supplementary Fig. 2b shows transport data in the same sample after it was re-exposed to oxygen (suspended gold is *p*-doping). In Supplementary Fig. 2a, T = 1.3 K, we observe Coulomb blockade diamonds corresponding to a SWCNT-QD whose charge occupation number is controlled by V_G. We note that the current (conductance) is much higher for electron occupation of the QD (V_G > 0 V) than for hole occupation of the QD (V_G < 0 V). This indicates that the contacts (SWCNT sections under the suspended gold) are *n*-doped. To acquire the data in Supplementary Fig. 2b, Device B was warmed up to 295 K and exposed to air to undo the effects of annealing. The transport data show a much higher conductance for hole rather than electron doping of the QD, indicating that the contacts are now *p*-doped. Supplementary Figure 2c highlights the difference between *n* and *p* doping of the contacts by showing I-V_G data extracted at V_B = 15 mV from Supplementary Figs. 2a-b.

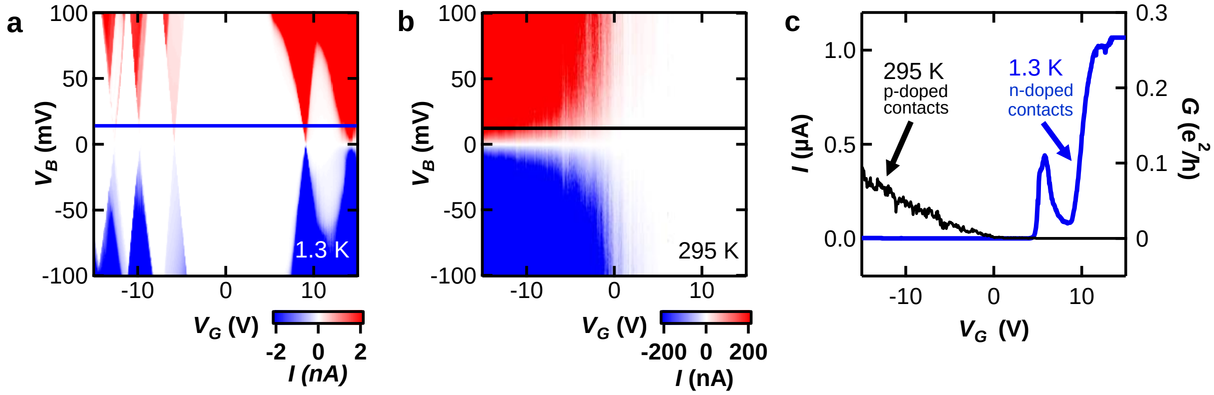

Supplementary Figure 2. Charge transfer from gold to SWCNTs: *n*-type to *p*-type **a-b** I-V_B-V_G data from Device B at 1.3 K and 295 K, showing *n*-doped and *p*-doped contacts to the SWCNT channel, respectively. The horizontal lines at V_B = 15 mV correspond to the data in **c** which show higher conductance for electrons at 1.3 K (blue data, annealed gold), and higher conductance for holes at 295 K (black data, oxygen-exposed gold).

Supplementary Note 2: Transport data and E_g measurements for all devices

We show differential conductance data for the five devices we report on in Supplementary Fig. 3. To determine the band gaps E_g of the tubes, we use data at the charge neutrality point (N = 0) corresponding to the tallest Coulomb blockade diamond. The band gap of a given nanotube can be determined from the following equation:

$$E_g = E_{\mathrm{add}}^{N=0} - E_c - \Delta \qquad \text{(S1)}$$

where E_g is the band gap, $E_{\mathrm{add}}^{N=0}$ is the height of the N = 0 diamond, E_c is the charging energy, and Δ is the single particle energy spacing. The contributions of E_c and Δ are often negligible [S3], but in our ultra-short devices they are too large to be ignored. These quantities are measured from the Coulomb diamonds in Supplementary Fig. 3. The measured values for E_g, $E_{\mathrm{add}}^{N=0}$, $E_{\mathrm{C}}^{N=-1}$ and Δ^h for all five devices are summarized in Supplementary Table 1 below. Details about the extraction of $E_{\mathrm{add}}^{N=0}$, $E_{\mathrm{C}}^{N=-1}$ and Δ^h are discussed in section 2.1.

Device	E_g (meV)	$E_{\mathrm{add}}^{N=0}$ (meV)	$E_{\mathrm{C}}^{N=-1}$ (meV)	Δ^h (meV)
A	28 ± 8	79 ± 8	39 ± 2	12 ± 1
B	270 ± 50	450 ± 50	120 ± 10	60 ± 10
C	190 ± 50	230 ± 50	36 ± 4	7 ± 4
D	250 ± 20	410 ± 20	140 ± 3	18 ± 6
E	170 ± 50	280 ± 50	109 ± 7	-

Supplementary Table 1: Band gap of the SWCNT in each device. We determine the band gap, E_g, from the parameters $E_{\mathrm{add}}^{N=0}$, $E_{\mathrm{C}}^{N=-1}$, and Δ^h extracted from Supplementary Fig. 3, and using Eqs. S2 and S4.

2.1 Extraction of $E_{add}^{N=0}$, $E_{C}^{N=-1}$ and Δ^h

The $E_{\mathrm{add}}^{N=0}$ values are the heights of the N = 0 diamonds in Supplementary Fig. 3. When the N = 0 diamond is too tall to be measured directly, we measure the slopes of the diamond's edges to extract its height. E_c and Δ are carrier type dependent, and we use the hole values because they show less dependence on the number of carrier inside the QD and better represent the N = *0* values. The value of $E_{\mathrm{C}}^{N=-1}$ is measured directly from the height of the N = -1 diamonds. In a SWCNT-QD showing a 4-fold degenerate diamond pattern (Device A), Δ is given by the difference between E_{add} (diamond height) for an N = 4n diamond and a N = 4n ± 1 diamond [S4], as shown in Eq. S2.

$$\Delta_{4fold} = E_{add}^{N=4n} - \frac{\left(E_{add}^{N=4n+1} + E_{add}^{N=4n-1}\right)}{2} \qquad \text{(S2)}$$

For Device A, using Eq. S2 we find, $\Delta^h = 12 \pm 1$ meV. Because this tube is nearly metallic, (small band gap), we use the single particle energy spacing for a metallic nanotube [S5],

$$L = \frac{hv_f}{2\Delta} \qquad \text{(S3)}$$

This gives a device length of 140 ± 10 nm. Considering that Eq. S3 is only an approximation for Device A, this length is in reasonable agreement with the SEM measured length of 111 ± 5 nm. It confirms that the channel length when the QD is doped with holes corresponds to the naked SWCNT channel visible in Figs. 1c and Supplementary Fig. 4.

When the channel of Device A is electron doped, its transport behavior no longer shows Coulomb Blockade (Supplementary Fig. 3a, right hand side), but rather Fabry-Pérot (FP) oscillations. The spacing of the first set of maxima around V_B = 0 gives the energy level spacing, and we find $\Delta^e = 5 \pm 1$ meV. Using Eq. S3 gives a FP cavity length of 330 ± 70 nm, which is vastly different from the naked SWCNT channel length (section 3) but matches the length of the suspended gold gates (Supplementary Fig. 1).

For 2-fold degenerate QD devices such as Device C and D, Δ is given by Eq. S4 below,

$$\Delta_{2fold} = E_{add}^{N=4n} + E_{add}^{N=4n+2} - \frac{\left(2E_{add}^{N=4n+1} + E_{add}^{N=4n-1} + E_{add}^{N=4n+3}\right)}{2} \quad \text{(S4)}$$

In Device C, we measure for hole and electron deltas: $\Delta^h \approx \Delta^e \approx$ 7 meV, and in Device D, $\Delta^h \approx \Delta^e \approx$ 18 meV. We note that the Δ 's for holes and electrons show that the QD channel length is the same for electrons and holes. We do not know the exact shape of the QD confinement potential, because the devices are very short and have significant contact doping. A rough estimate of the device length can be calculated using the measured Δ and a harmonic potential [S3], as shown in Eq. S5. These lengths are in agreement with the more precise measurements presented in section 3.

$$\Delta = \hbar\sqrt{\frac{4E_g}{m_{eff}L^2}}, \qquad m_{eff} = \frac{\left[\left(hv_f/2L\right)^2 + \left(E_g/2\right)^2\right]^{3/2}}{\left(v_f E_g/2\right)^2} \quad \text{(S5)}$$

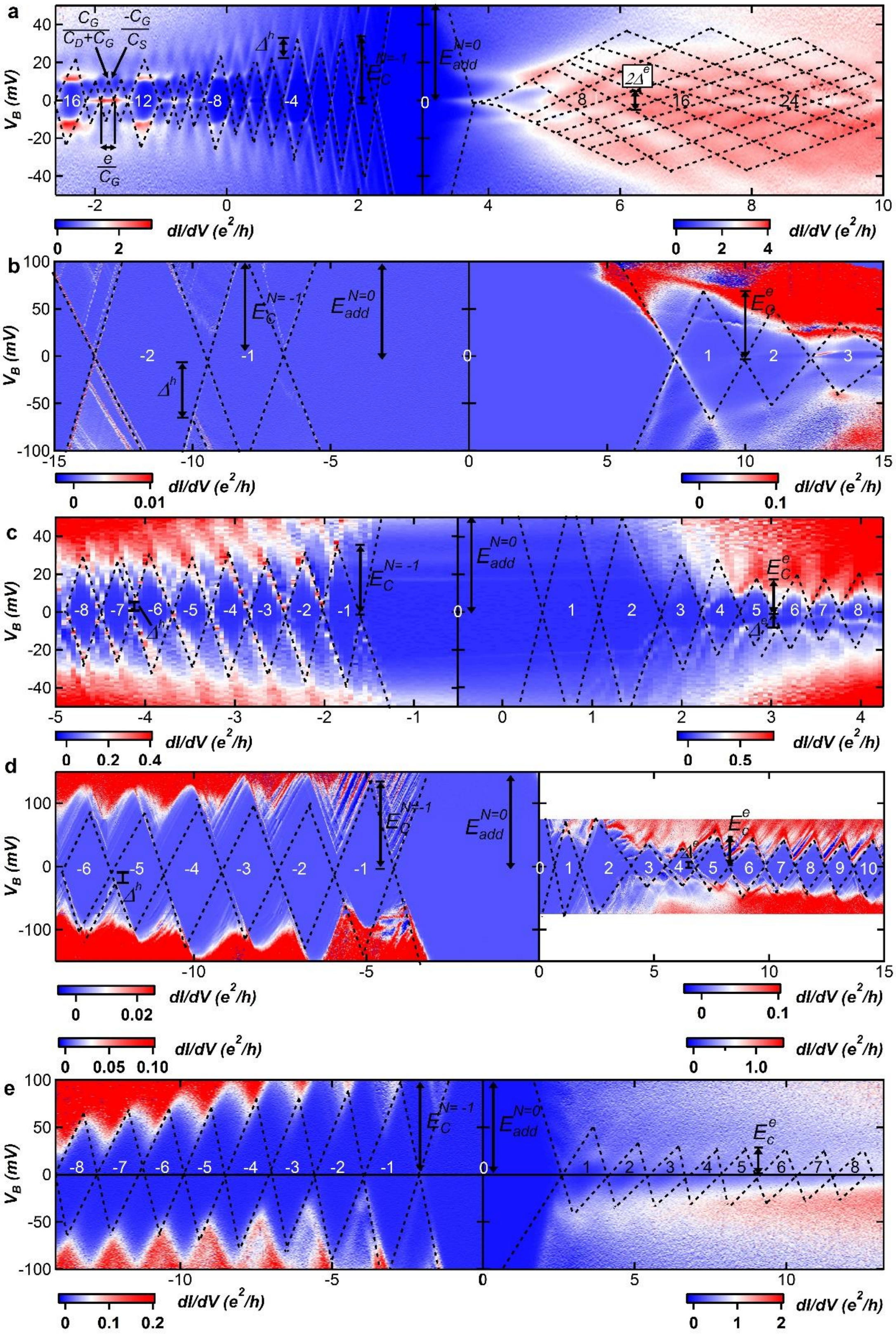

Supplementary Figure 3. dI/dV-V_B-V_G data for each device. **a-e** Devices A, B, C, D, and E respectively. T = 1.3 K for Devices A, B, C, T = 4 K for Device D, and T = 50 K for Device E.

Supplementary Note 3: Channel lengths

To analyze our data, it is important to accurately determine the lengths of our ultra-short nanotube channels. We combine information from SEM images and Coulomb diamonds to determine the length of our SWCNT quantum dots.

3.1 Naked SWCNT length: SEM measurements

We captured high resolution SEM images of our devices at nearly all stages of their fabrication. Note that the naked channels were only imaged after the transport data were acquired. In Supplementary Fig. 4, SEM images of each device are shown on a separate row. The images in the left column are superpositions of SEM images before and after deposition of the gold contacts. These before and after deposition images were aligned using the positions of protruding SWCNTs and alignment markers. Using the positions of the nanotubes before metal deposition, we can locate the tube positions after EM (right column). We drew dashed lines on Supplementary Fig. 4 to indicate the SWCNT positions. From the right column images, we measured the lengths of the naked SWCNT channels, L_{SEM}, and summarize the results in Supplementary Table 2.

Device	L_{SEM} (nm)	L_G (nm)
A	111 ± 5	102 ± 5
B	14 ± 3	7 ± 5
C	42 ± 7	46 ± 8
D	16 ± 4	13 ± 5
E	24 ± 8	15 ± 5

Supplementary Table 2. Channel lengths. L_{SEM} and L_G for all devices.

3.2 Naked SWCNT length: gate capacitance (C_G) measurement

From the widths of the Coulomb diamonds (Supplementary Fig. 3), we determined the gate capacitance of each device using $C_G = e/\Delta V_G$, where ΔV_G is the width of the odd Coulomb diamonds and *e* is the elementary charge. The capacitance of a back-gated SWCNT devices is modeled as a wire over a plane using:

$$\frac{C_G}{L_G} = \frac{2\pi\varepsilon}{\cosh^{-1}\left(\frac{t}{r}\right)} \tag{S6}$$

where L_G is the length of the wire, ε is the permittivity of the insulator, *t* is the thickness of the insulator, and *r* is the radius of the wire. In our devices, the dielectric spacer is made of two thin films in series: vacuum and SiO_2. The relevant parameters are shown in Supplementary Table 3. The gate capacitance is given by [S6]:

$$\frac{C_G}{L_G} = \frac{2\pi\varepsilon_{ox}}{\frac{\varepsilon_{ox}}{\varepsilon_{vac}} * \cosh^{-1}\left(\frac{t_{vac}}{r}\right) + \cosh^{-1}\left(\frac{t_{vac}+t_{ox}}{r+t_{vac}}\right)} \tag{S7}$$

Where ε_{ox}, ε_{vac} are the permittivities, and t_{ox}, t_{vac} the thickness of the oxide and vacuum spacers respectively.

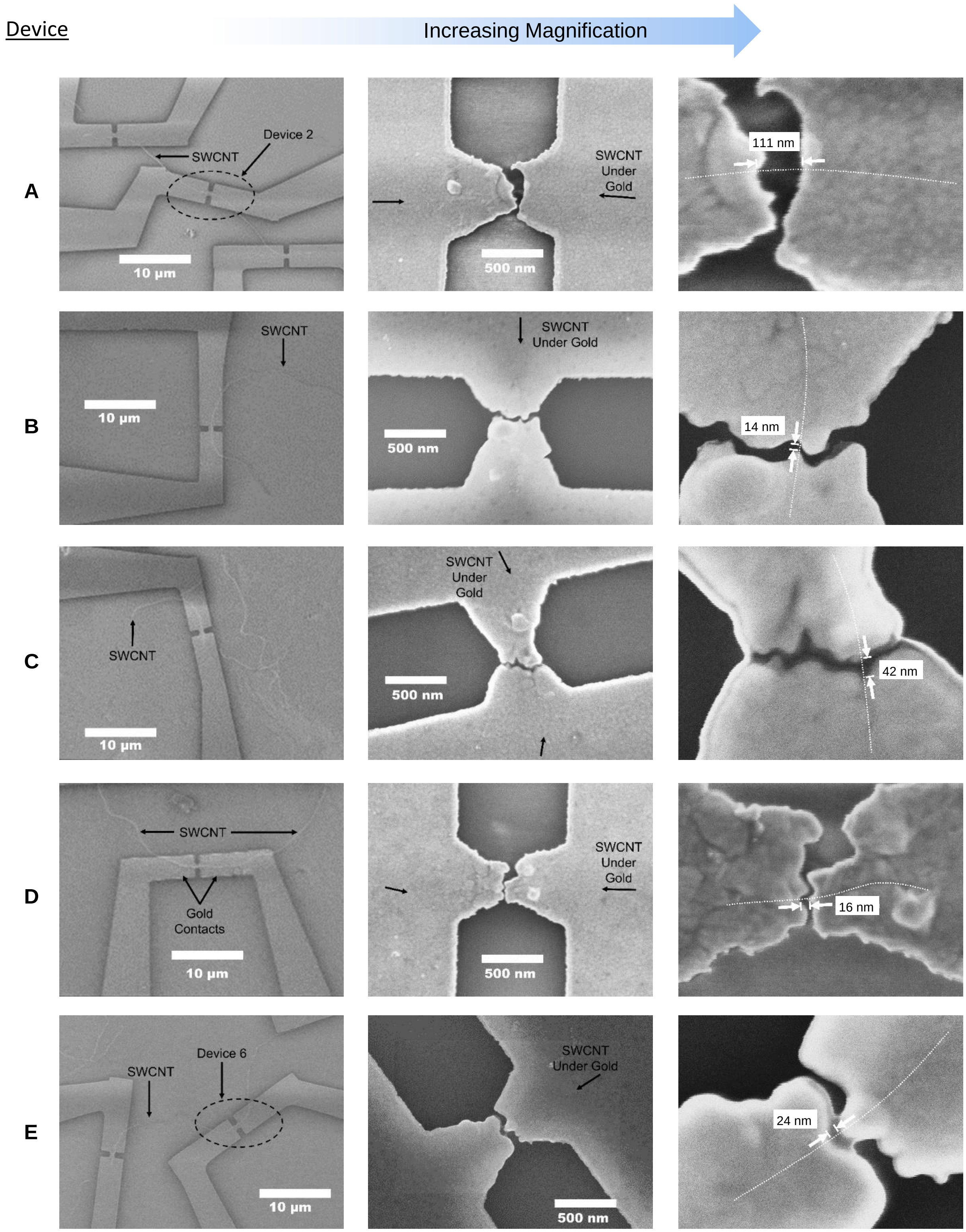

Supplementary Figure 4. SEM images of each device. The device's names are on the left, and the magnification of the images increases from the left to the right column. The rightmost images show the SWCNT channels of each device (the dashed lines are guides to the eye).

We measured the oxide thickness using ellipsometry after the buffered oxide etch, and measured the nanotube diameter using AFM. The relevant device parameters for capacitance modeling are summarized in Supplementary Table 3. Using these values and Eq. S7, we calculated the electrostatic lengths, L_G, of the SWCNT-QD which are summarized in Supplementary Table 2 above. We note that AFM tends to underestimate SWCNT diameters, and thus the measured radius values, *r*, should be considered as lower bounds. However, the calculated values of L_G depend only very weakly on precise values of *r*.

Device	C_G (aF)	t_{ox} (nm)	t_{vac} (nm)	r (Å)
A	0.59 ± 0.04	202	126	≈ 3
B	0.05 ± 0.01	170	134	≈ 3
C	0.41 ± 0.07	169	139	≈ 13
D	0.11 ± 0.01	172	135	≈ 8
E	0.12 ± 0.01	170	134	≈ 5

Supplementary Table 3. Gate capacitance parameters (measured).These parameters are used in Eq. S7 to determine the electrostatic lengths, L_G, of the SWCNT QDs.

The gate capacitance, C_G, was extracted by averaging over all odd diamonds for $|V_G| >$ 6 V, where the widths are roughly constant and unaffected by the quantum energy level spacing Δ. Comparing the values of L_G to L_{SEM} in Supplementary Table 2, we see a strong agreement. We can estimate the length of the two *p-n* junctions forming at the ends of the naked channel as the difference between L_{SEM} and L_G. We label the length of one *p-n* junction as L_{pn}. While the errors on individual L_{SEM} and L_G are too large to get a good measurement of L_{pn} for a single device, by combining the measurements in all 5 devices we find L_{pn} = 3 ± 1 nm.

Supplementary Note 4: Homojunctions and tunnel barriers

Using Anderson's rule [S7], we draw the approximate shape and height of the barriers forming at the homojunctions between the gold-covered and bare SWCNT suspended sections (Supplementary Fig. 5 below, and main text Figs. 2a,b and 3a,b). There are two needed input parameters, which we do not directly measure, to draw these diagrams: $\Delta E_{F,1}$ and $\Delta\chi$. The former is the displacement of the SWCNT's Fermi level underneath the annealed-gold film. The exact amount of doping $\Delta E_{F,1}$ varies depending on the crystalline orientation of the gold as well as the quality of annealing (oxygen content). The relevant values for our devices are reported to range from 0.05 to 0.2 eV [S8-S10], and we used a median value of 0.12 eV to draw our band diagrams. The gold film also modifies the nanotube's electron affinity by $\Delta\chi$. This shift is expected to be around 0.03 - 0.05 eV [S9, S10], and we used 0.05 eV to draw the bands. We note that our conclusions are valid over a broad range of values for $\Delta E_{F,1}$ and $\Delta\chi$; and the transport data presented and discussed in the main text offer strong support for the band alignments in Figs. 2, 3 and Supplementary Fig. 5.

The other quantities needed to draw the band diagrams are E_g (measured in section 2), and the Fermi level displacement $\Delta E_{F,2}$ in the naked channel which was tuned experimentally using V_G to match $\Delta E_{F,2} = \pm E_g/2$.

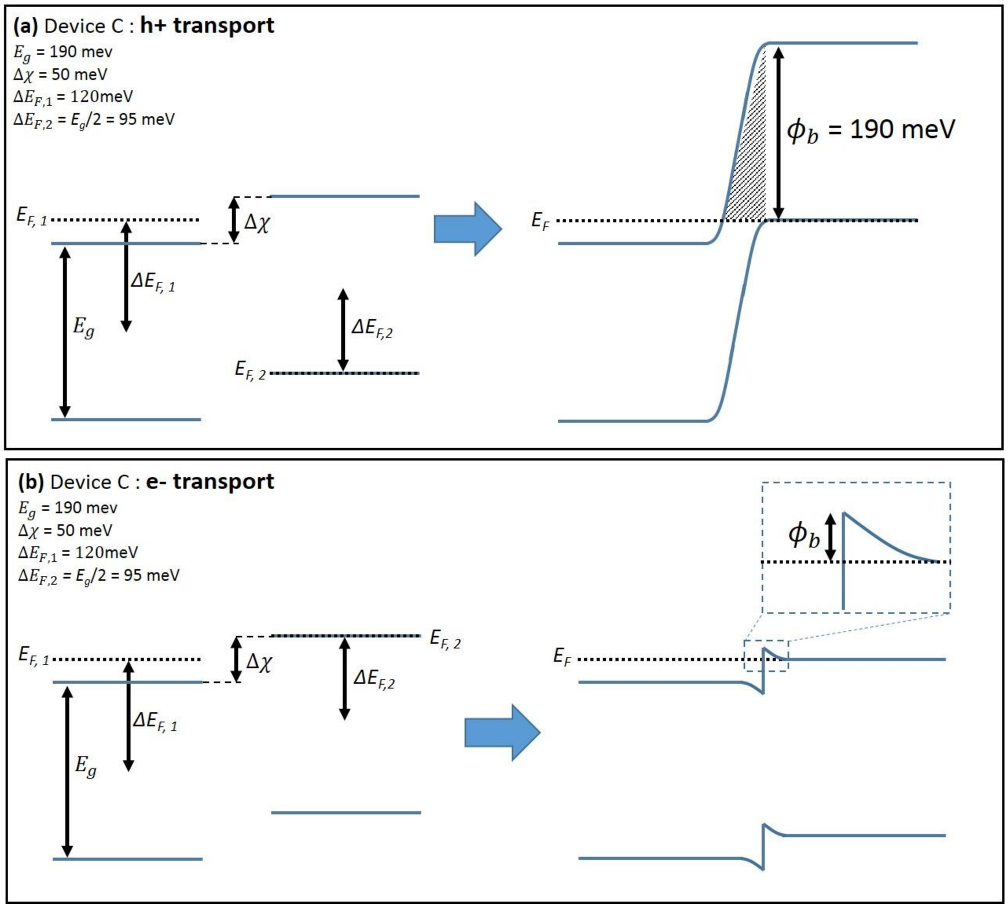

Supplementary Figure 5. Schematic of the barriers between the naked and gold-covered SWCNT sections in Device C. **a** when the naked channel is hole**-**doped and **b** electron-doped. The left hand side of each panel shows the bands in the two SWCNT sections (gold-covered and naked) before they are allowed to equilibrate. The naked channel is doped using the back gate voltage, and there is a small electron affinity difference of $\Delta\chi$ between the two SWCNT sections. The right hand side of each panel show the junctions when the two sections equilibrate. The bands are aligned using Anderson's rule [S7].

We note that the asymmetric barrier heights for holes and electrons (Supplementary Table 4) correctly predicts the sign of the *e-h* charging energy asymmetry (Supplementary Table S5) for all devices. It also predicts correctly the presence or absence of a barrier in all devices, and the qualitative magnitude of the asymmetry is correctly captured. Our conclusions are insensitive to the quantitative details of the barrier shapes and heights.

Devices	$\boldsymbol{E_g}$ (meV)	$\boldsymbol{\phi_b{}^e}$ (meV)	$\boldsymbol{\phi_b{}^h}$ (meV)
A	28	0	28
B	270	27.5	270
C	190	12.5	190
D	250	27.5	250
E	170	7.5	170

Supplementary Table 4. Estimated barrier heights in each reported device. The barrier heights are $\phi_b{}^e$ and $\phi_b{}^h$ for e^- doping and h^+ doping of the channel, respectively. The qualitative asymmetries of the barrier heights agree with the observed charging energy asymmetries (Table S5) for all devices.

Supplementary Note 5: Electron-hole charging energy asymmetry

We quantify the asymmetry between the charging energy of holes, E_C^h, and electrons, E_C^e, in the five reported devices using the following procedure. We first extract the addition energy, E_{add}, versus *N* for all five devices from the data in Supplementary Fig. 3 and show these numbers in Supplementary Fig. 6. The black data in Supplementary Fig. 6 show E_{add}^h while the red data show E_{add}^e. We then interpolate (dashed lines) E_{add} using only the odd-*N* values, because $E_{add} = E_C$ for odd *N*. These interpolations show the values of E_C^h and E_C^e vs *N* (except for E_C^e in Device A which must be calculated using an open-QD model [S11]) . We observe that E_C first drops quickly with increasing *N* and then stabilizes. To avoid this strong *N* dependence at low *N*, we extract E_C^h and E_C^e at *N* = 5 for all devices, except Device B where we use *N* = 1 since the data set does not include *N* = 5. We calculate the relative charging energy asymmetry, $\eta_{e-h} = \frac{E_C^h}{E_C^e}$, as shown in Supplementary Table 7.

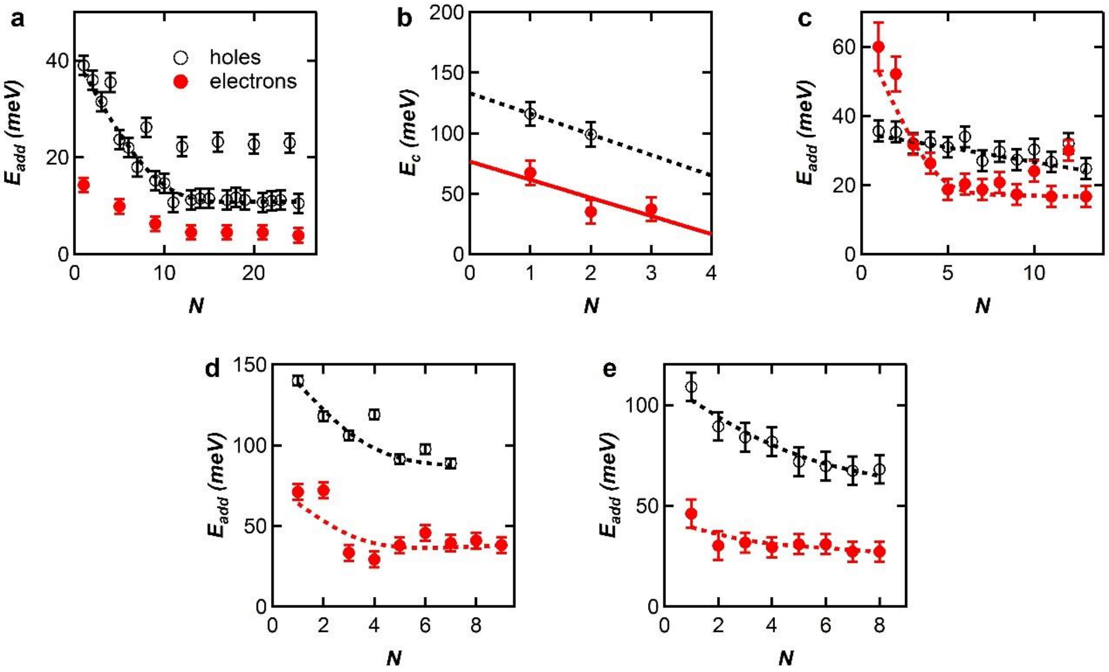

Supplementary Figure 6: E_{add} and E_C for holes and electrons vs. N in each device. **a-e** Data from Devices A-E. Red and black data correspond to electrons and hole doping of the naked SWCNT channels. The dashed lines are interpolations of odd-*N* data, except in panel **b** where they are linear fits to the data. The relative charging asymmetry η_{e-h} is calculated from the interpolations at *N* = 5 (*N* = 1 for **b**).

We verified that the η_{e-h} asymmetry is not strongly dependent on the details of the analysis. For instance we find almost identical η_{e-h} if we compare the charging energies corresponding to diamonds located around V_G = ± 6V instead of at *N* = 5.

Device	η_{e-h} $(N = 5)$
A	$\gtrsim 100$
B	2.6
C	1.5
D	2.1
E	2.5

Supplementary Table 5. Electron-hole charging energy asymmetry ratios $\eta_{e-h} = E_C^h/E_C^e$

Supplementary References

[S1] W. Jeong, K. Kim, Y. Kim, W. Lee, and P. Reddy, *Sci. Rep.* **4**, 4975 (2014).

[S2] S. Heinze, *et al.*, *Phys. Rev. Lett.* **89**, 106801 (2002).

[S3] P. Jarillo-Herrero, *et al.*, *Nature* **429**, 391 (2004).

[S4] E. A. Laird, *et al*., *Rev. Mod. Phys.* **87**, 703 (2015).

[S5] W. Liang, *et al.*, *Nature* **411**, 665 (2001).

[S6] J. Island, V. Tayari, A. McRae, A. R. Champagne, *Nano Lett.* **12**, 4564 (2012).

[S7] J. H. Davies, The Physics of low-dimensional semiconductors, Cambridge (1998).

[S8] X. D. Cui, M. Freitag, R. Martel, L. Brus, and P. Avouris, *Nano Lett.* **3**, 783 (2003).

[S9] P. A. Khomyakov, *et al.*, *Phys. Rev. B.* **79**, 195425 (2009).

[S10] M. Hasegawa and K. Nishidate, *Phys. Rev. B* **84**, 155435 (2011).

[S11] H. I. Jorgensen, *et al.*, *Phys. Rev. B* **79**, 155441 (2009).
